# The impact of training on data from genetically-related lines on the accuracy of genomic predictions for feed efficiency traits in pigs

**DOI:** 10.1186/s12711-020-00576-0

**Published:** 2020-10-07

**Authors:** Amir Aliakbari, Emilie Delpuech, Yann Labrune, Juliette Riquet, Hélène Gilbert

**Affiliations:** GenPhySE, Université de Toulouse, INRAE, 31326 Castanet-Tolosan, France

## Abstract

**Background:**

Most genomic predictions use a unique population that is split into a training and a validation set. However, genomic prediction using genetically heterogeneous training sets could provide more flexibility when constructing the training sets in small populations. The aim of our study was to investigate the potential of genomic prediction of feed efficiency related traits using training sets that combine animals from two different, but genetically-related lines. We compared realized prediction accuracy and prediction bias for different training set compositions for five production traits.

**Results:**

Genomic breeding values (GEBV) were predicted using the single-step genomic best linear unbiased prediction method in six scenarios applied iteratively to two genetically-related lines (i.e. 12 scenarios). The objective for all scenarios was to predict GEBV of pigs in the last three generations (~ 400 pigs, G7 to G9) of a given line. For each line, a control scenario was set up with a training set that included only animals from that line (target line). For all traits, adding more animals from the other line to the training set did not increase prediction accuracy compared to the control scenario. A small decrease in prediction accuracies was found for average daily gain, backfat thickness, and daily feed intake as the number of animals from the target line decreased in the training set. Including more animals from the other line did not decrease prediction accuracy for feed conversion ratio and residual feed intake, which were both highly affected by selection within lines. However, prediction biases were systematic for these cases and might be reduced with bivariate analyses.

**Conclusions:**

Our results show that genomic prediction using a training set that includes animals from genetically-related lines can be as accurate as genomic prediction using a training set from the target population. With combined reference sets, accuracy increased for traits that were highly affected by selection. Our results provide insights into the design of reference populations, especially to initiate genomic selection in small-sized lines, for which the number of historical samples is small and that are developed simultaneously. This applies especially to poultry and pig breeding and to other crossbreeding schemes.

## Background

Given the large economic impact of feed efficiency in the swine industry, its evaluation requires accurate estimation of breeding values (BV) and selection of animals [1]. The most commonly used criterion to measure feed efficiency in livestock species is feed conversion ratio (FCR) and is defined as feed intake per unit of live weight gain [2]. However, in 1963, residual feed intake (RFI) was introduced in cattle as an alternative criterion for feed efficiency [3]. In general, FCR and RFI are highly genetically correlated [4]. Nevertheless, selection of animals based on FCR can be accompanied by undesirable correlated responses in other traits such as appetite [5, 6], whereas selection for RFI is almost independent of these traits since RFI is feed intake adjusted for production trait by linear regression. Due to the high cost of measuring daily feed intake (DFI), and thus RFI and FCR [7], fewer phenotypic records are available, which reduces the accuracy of selection. Genomic selection has the potential to improve pig feed efficiency in some populations [8, 9]. Recent advances in genomic evaluation methodologies, such as single-step genomic best linear unbiased prediction (ssGBLUP), enable more accurate evaluations in small populations. The ssGBLUP combines phenotypic, genotypic, and pedigree information in a single genomic evaluation of animals [10–13]. The number of animals in the reference population has been shown to affect the accuracy of genomic predictions [14]. Multi-breed or admixed genomic evaluations have been proposed to increase the number of animals in reference sets for small populations [15], resulting in increases in prediction accuracy in some cases [16]. A study on multi-breed genomic evaluation using real data from Holstein and Jersey bulls showed that using a combined reference population resulted in comparable accuracies of genomic estimated breeding values (GEBV) in purebred validation sets, or exceeded that achieved with a purebred reference population of the same breed [17]. Adding a smaller population, i.e. Brown Swiss, to a reference population of Holstein and Jersey bulls resulted in slight increases in accuracy of predictions when breeds were considered as a single, joint population, while slight increases in accuracy were also observed if the breeds were treated as genetically-related traits [18]. Simulation studies with mixed reference populations also showed increases in prediction accuracy. A simulation study on genomic prediction across multiple populations in cattle showed that adding relatively few individuals from another population to a training set substantially increased the accuracy of predictions in the first population, regardless of the heritability (h^2^) or marker density [19]. Another simulation study reported that genomic predictions using a combined versus a single reference population increased the accuracy of genomic predictions by 25%, with traits with a lower heritability benefiting more from the combination of populations [20]. However, using a combined reference population can be challenging if relationships between populations are absent: allele frequencies at the marker and/or causal loci, or causal variants themselves, can differ between populations, [15, 16]. Another limitation for across-breed genomic prediction is the inconsistency of linkage disequilibrium (LD) between markers and quantitative trait loci (QTL) between breeds, which is one of the assumptions of most genomic prediction models [17].

Given the presence of (ancestral) relationships between animals and the greater consistency of LD between genetically-related lines within a breed than between breeds that have been separated for decades, using a multi-line reference population may be more beneficial than using a multi-breed reference population [16]. However, the changes in allele frequency since separation of the lines may still represent a challenge for using a multi-line reference population [21]. To the best of our knowledge, the use of a multi-line genomic evaluation strategy in small, related lines using real data has not been studied, in spite of the existence of numerous related lines worldwide. Our hypothesis was that, in small porcine populations with few available ancestral samples, i.e. for which it is not possible to build large reference populations, including information from a genetically-related line in the training population could provide similar prediction accuracies as a within-line training population. Therefore, we explored reference populations with different structures that combined data from two lines that descended from a common origin, and compared the prediction accuracy obtained with that obtained when only information from the target line was used for training.

## Methods

### Population and data structure

The data were collected during a selection experiment that was conducted at INRAE (UE GenESI, Surgères, France, 10.15454/1.5572415481185847E12) on French Large White pigs. Two lines were established by nine generations of divergent selection for RFI from 2000 to 2015 [22]. The G0 generation resulted from the mating of 30 boars and 30 gilts from generation F0 using artificial insemination. Among the G0 animals, 116 boar candidates for selection from all 30 litters were tested for RFI to select six extreme founder boars for each line (LRFI: low RFI, and HRFI: high RFI). The two lines were initiated by mating the selected boars to about 35 random G0 gilts per line. Inbreeding was minimized at each generation. The development of each line continued with the selection of six boars out of 96 tested candidates in each generation from G1 to G9. In each generation, at least one additional parity was produced to evaluate correlated responses to selection for production traits on both females and castrated males (henceforth referred to as response animals). Selection candidates were evaluated for RFI from 35 to 95 kg of body weight (BW), and response animals were evaluated from 10 weeks of age until slaughter (105 kg BW until G5 and 115 kg BW from G6 onwards). Animals were raised in four pens per batch and at least four batches per generation. Test pens were equipped with single-place electronic feeders ACEMA64 (ACEMO, France). Animals were offered ad libitum access to a pelleted diet based on cereals and soya bean meal containing 10 MJ net energy (NE)/kg and 160 g CP/kg, with a minimum of 0.80 g digestible Lys/MJ NE. In each generation, boars were selected based on a fixed RFI selection index that was established from pre-computed phenotypic correlations between DFI (g/day) and average daily gain (ADG, g/day) between 35 and 95 kg BW, and live backfat thickness (BFT, mm) at 95 kg BW [23], as RFI = DFI − 1.06 × ADG − 37 × BFT. The average metabolic BW (AMBW) was the same for all selection candidates and therefore excluded from the selection index equation. Selection candidates had records for feed intake, body weight, and live body composition traits. In addition to these phenotypes, gilts and castrated males had records for carcass composition traits [23]. For the present study, RFI, FCR, DFI, ADG and BFT were analyzed. These traits were available for both selection candidates and response animals. The number of observations for the five traits for each line are in Table 1. RFI of selection candidates was computed between 35 and 95 kg BW as the residual of a multiple linear regression of DFI on the traits included in the selection index. For gilts and castrated males from the correlated response batches, RFI was estimated from 10 weeks of age to slaughter as the residual of a multiple linear regression of DFI on AMBW, ADG from 10 weeks of age to slaughter, carcass BFT (carcBFT), and lean meat content (LMC; computed from cut weights) at slaughter. AMBW was included to account for maintenance requirements and the other traits were included to account for production requirements. [22]. Fixed effects included in the regression model to compute RFI of response animals were sex, pen size, contemporary group and BW at the beginning of the test. Complete pedigree information was collected from F0 to G9, plus up to 10 generations of ancestors, and contained 7046 animals (Table 1).

**Table 1:** Numbers of animals in the pedigree and data structure

	Ancestors	F0	G0	HRFI										
				G0	G1	G2	G3	G4	G5	G6	G7	G8	G9	Total
Pedigree	159	67	104	48	216	297	277	260	270	795	474	292	280	3209
Pedigree only				1	2	89	78	62	68	352	149	5	0	806
Pedigree and genotype only				41	41	42	44	36	47	40	35	42	91	459
ADG														
Phenotype only				0	167	160	149	156	149	304	194	148	93	1520
Phenotype and genotype				6	6	6	6	6	6	71	73	66	92	338
Missing				0	0	0	0	0	0	28	23	31	4	86
BFT														
Phenotype only				0	167	160	149	156	149	237	176	62	84	1340
Phenotype and genotype				6	6	6	6	6	6	71	73	66	92	338
Missing				0	0	0	0	0	0	95	41	117	13	266
DFI														
Phenotype only				0	166	160	149	156	149	263	182	138	93	1456
Phenotype and genotype				6	6	6	6	6	6	71	73	66	92	338
Missing				0	1	0	0	0	0	69	35	41	4	150
FCR														
Phenotype only				0	166	160	148	156	149	263	182	138	93	1455
Phenotype and genotype				4	6	6	6	6	6	71	73	66	92	336
Missing				2	1	0	1	0	0	69	35	41	4	153
RFI														
Phenotype only				0	164	159	146	156	143	185	147	56	80	1236
Phenotype and genotype				6	6	6	6	6	6	71	73	66	92	338
Missing				0	3	1	3	0	6	147	70	123	17	370

	Ancestors	0	G0	LRFI										
				G0	G1	G2	G3	G4	G5	G6	G7	G8	G9	Total
Pedigree	159	67	104	46	203	303	314	327	357	826	481	344	280	3481
Pedigree only				0	1	98	100	107	130	337	132	8	0	913
Pedigree and genotype only				40	35	40	41	43	43	48	55	48	93	486
ADG														
Phenotype only				0	161	159	167	171	178	359	211	203	95	1704
Phenotype and genotype				6	6	6	6	6	6	74	73	74	90	347
Missing				0	0	0	0	0	0	8	10	11	2	31
BFT														
Phenotype only				0	161	159	167	171	178	284	206	105	86	1517
Phenotype and genotype				6	6	6	6	6	6	74	73	74	90	347
Missing				0	0	0	0	0	83	15	109	1	1	218
DFI														
Phenotype only				0	160	159	167	171	178	316	206	194	95	1646
Phenotype and genotype				6	6	6	6	6	6	74	73	74	90	347
Missing				0	1	0	0	0	0	51	15	20	2	89
FCR														
Phenotype only				0	159	159	167	171	178	316	208	195	95	1648
Phenotype and genotype				6	6	6	6	6	74	73	74	90		347
Missing				0	2	0	0	0	0	51	13	19	2	87
RFI														
Phenotype only				0	160	158	161	171	173	230	165	101	80	1399
Phenotype and genotype				6	6	6	6	6	6	74	73	74	90	347
Missing				0	1	1	6	0	5	137	56	113	17	336

*HRFI* high RFI line, *LRFI* low RFI line, *Ancestors* animals before the base generation, *F0* base generation, *G0 to G9* generations of selection 0 to 9, *RFI* residual feed intake, *ADG* average daily gain, *FCR* feed conversion ratio, *DFI* daily feed intake, *BFT* backfat thickness

### Combining and standardizing traits

Preliminary analyses on the five traits showed high genetic correlations between similar traits measured in selection candidate and response animals (> 0.80 ± 0.11, except 0.75 ± 0.08 between live BFT and carcass BFT). Therefore, to increase the amount of information, corresponding traits in selection candidate and response animals were combined for further analyses. Since animals differed in age and BW when measurements were taken, for each trait, records from selection candidates were standardized to the variance of the corresponding trait in the response animals as:

$$y_{Rij} = \frac{{y_{sij} }}{{\sigma_{si} }}\sigma_{Ri}$$

where $y_{Rij}$ is the standardized trait $i$ ($i$ = 1,… 5) for selection candidate *j,* $y_{sij}$ is the record of trait $i$ measured on animal $j$, $\sigma_{si}$ is the phenotypic standard deviation of trait $i$ measured on selection candidates, and $\sigma_{Ri}$ is the phenotypic standard deviation of trait $i$ measured on females and castrated males in the response batches. Descriptive statistics of these traits are in Table 2.

**Table 2:** Descriptive statistics of the data for the studied traits in the HRFI and LRFI lines

Line	Trait	Number of records	Minimum	Maximum	Average	Coefficient of variation
HRFI	ADG	1868	0.44	1.07	0.76	11.03
	BFT	1687	9.67	49.27	27.33	26.62
	DFI	1802	1.37	3.20	2.18	12.54
	FCR	1799	2.13	3.81	2.8	9.26
	RFI	1581	− 0.29	0.86	0.05	–
LRFI	ADG	2053	0.45	1.06	0.76	10.69
	BFT	1866	10.00	44.63	26.45	24.60
	DFI	1995	1.05	2.92	2.01	12.91
	FCR	1997	1.72	3.70	2.60	9.11
	RFI	1748	− 0.56	0.46	− 0.04	–

*HRFI* high RFI line, *LRFI* low RFI line, *ADG* average daily gain (kg/day), *BFT* backfat thickness (mm), *DFI* daily feed intake (kg/day), *FCR* feed conversion ratio (kg/kg), *RFI* residual feed intake (kg/day)

### Single nucleotide polymorphism (SNP) genotyping data and imputation

SNP genotyping data were available for all selected boars and their mates from G0 to G9, additional pigs from response batches of G6 to G8, and all selection candidates in G9. In total, 1647 animals had SNP genotypes, of which 286 animals were genotyped with the Porcine SNP60v2 BeadChip (Illumina) (64,232 SNPs) and 1361 animals with the GGP Porcine HD Array (Illumina) (68,516 SNPs). Genotype quality control excluded SNPs with a call rate lower than 95%, individuals with a call rate lower than 90%, SNPs that were not in Hardy–Weinberg equilibrium (p < 10^−10^), SNPs with a minor allele frequency lower than 0.01, and individuals with parent–offspring incompatibility (e.g., opposite homozygotes) with at least one parent. The PLINK software was used for SNP and individual genotype quality control [24]. SNPs on the sex chromosomes were removed. After quality control of each SNP chip dataset, the SNPs present in each panel were imputed to the alternative panel using the FImpute software [25] in a single step. The two SNP chips shared 42,800 SNPs. The number of genotyped animals retained after imputation was 1643, and the final genotype dataset contained 64,233 informative SNPs. Thus, all animals had equal genotypic information. Genotypes were coded as 0, 1, or 2 for later calculation of the genomic relationship matrix. The number of animals with genotype data per generation and line is in Table 1.

### Model and analyses

Predictions obtained with BLUP are based on the assumption of no genetic differences between subpopulations [26, 27]. Therefore, to account for selection in our dataset, all genetic and genomic analyses were carried out with bivariate approaches, i.e. the five other traits were individually paired with the selection index in two-trait model analyses. By including the selection criterion, the analyses of other traits are conditioned based on all the information that was used for selection [28–30].

Preliminary analyses were carried out using a general linear model in R (glm procedure) to evaluate the significance (p < 0.05) of fixed environmental sources of variation. The significant fixed factors included pen size (5 levels: 8, 9, 10, 11, 12 pigs per pen), herd of birth (2 levels), sex (3 levels), and contemporary groups (CG, 99 levels). BW at slaughter was fitted in the model as a covariate only for BFT. CG were defined as animals born in the same week and raised in the same enclosure. Litter was fitted as a random environmental source of variation and its significance at the 5% level was determined using a likelihood ratio test.

The genetic analyses were performed using the AIREMLF90 and BLUPF90 software [31] for the BLUP and ssGBLUP methods, respectively. Prior to ssGBLUP evaluations, the variance components of the traits were obtained using the restricted maximum likelihood algorithm implemented in AIREMLF90. These analyses were performed using all available data and only the full pedigree relationship matrix (${\mathbf{A}}$). Variance components were estimated with the bivariate animal mixed model as follows:

$${\mathbf{y}} = {\mathbf{Xb}} + {\mathbf{Z}}_{1} {\mathbf{a}} + {\mathbf{Z}}_{2} {\mathbf{l}} + {\mathbf{e}}$$

where ${\mathbf{y}}$ is the vector of observations for the index and one of the five studied traits, ${\mathbf{b}}$ is the vector of fixed effects (described above), ${\mathbf{a}}$ is the vector of additive genetic effects, ${\mathbf{l}}$ is the vector of litter effects, and ${\mathbf{e}}$ is the vector of random residuals. ${\mathbf{X}}$, ${\mathbf{Z}}_{1}$ and ${\mathbf{Z}}_{2}$ are the incidence matrices for ${\mathbf{b}}$, ${\mathbf{a}}$, and ${\mathbf{l}}$, respectively. Distributions assumed for the random terms are ${\mathbf{a}}\sim N \left( {{\mathbf{0}}, {\mathbf{G}}_{0} \otimes {\mathbf{A}}} \right)$, ${\mathbf{l}}\sim N\left( {{\mathbf{0}}, {\mathbf{R}}_{{\mathbf{l}}} \otimes {\mathbf{I}}} \right)$, ${\mathbf{e}}\sim N\left( {{\mathbf{0}}, {\mathbf{R}}_{{\mathbf{e}}}\otimes {\mathbf{I}}} \right)$, where ${\mathbf{G}}_{0}$ is a $2 \times 2$ symmetric (co)variance matrix of direct additive genetic effects, and ${\mathbf{R}}_{{\mathbf{l}}}$ and ${\mathbf{R}}_{{\mathbf{e}}}$ are $2 \times 2$ symmetric (co)variances matrices of litter and residual effects, respectively. ${\mathbf{I}}$ denotes the identity matrix.

Genomic breeding values were estimated using ssGBLUP with the same models in the BLUPF90 software, with the previously estimated (co)variances and using the ${\mathbf{H}}$ matrix, which is a combined relationship matrix of the ${\mathbf{A}}$ matrix and marker-based relationship matrix (${\mathbf{G}}$) of genotyped animals [10, 12]. The ${\mathbf{G}}$ matrix was constructed and scaled by $2\sum \left\{ {p_{i} \left( {1 - p_{i} } \right)} \right\}$, where $p_{i}$ is the frequency of the second allele at locus $i$, following VanRaden [32]. Computation of the ${\mathbf{H}}$ matrices used outputs of BLUPF90 (${\mathbf{G}}$) and the full ${\mathbf{A}}$ matrix, which was obtained using the AGHmatrix R package [33]. In all scenarios, ${\mathbf{G}}$ had similar average diagonal elements as the pedigree relationship matrix for the genotyped animals (${\mathbf{A}}_{22}$).

### Scenarios

**Fig. 1:**
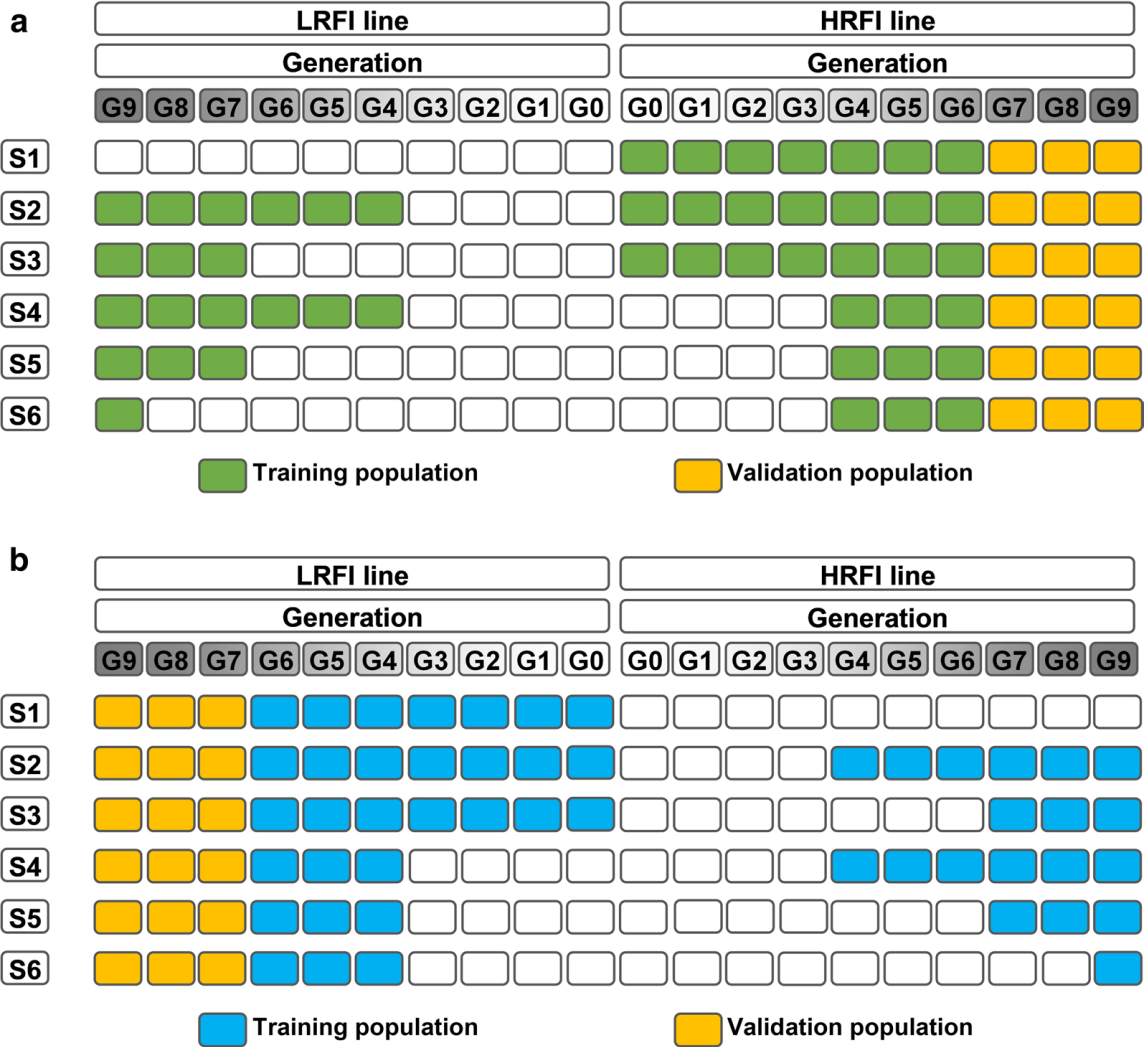
Design of scenarios to predict validation animals in HRFI (**a**) and LRFI (**b**) lines

Two symmetric series of six scenarios, one for each line, were defined for genomic prediction. An overview of the scenarios is shown in Fig. 1. In all scenarios, genotyped animals of the last three generations (G7 to G9, 433 pigs for the LRFI and 399 pigs for the HRFI line) were considered for validation in a given line (target line), and their information was removed from the training dataset.

The training sets were structured based on which generations and line were used. Scenario 1 comprised only animals from the target line and was the control scenario since it represented a routine genomic prediction design where all data would be available from the same line. All other scenarios were compared to this control scenario to evaluate which combination of training populations from the two lines achieved a prediction accuracy similar to the control scenario. Scenarios 2 and 3 included the training set of scenario 1 and in addition, either the animals from G4 to G9 (scenario 2), or G7 to G9 (scenario 3) of the other line.

For scenarios 4 to 6, animals from the target line in the training set were limited to the three generations nearest to the validation set (G4 to G6). In scenarios 4 and 5, the contribution to the training set of the animals from the other line was as in scenario 2 (G4 to G9) and scenario 3 (G7 to G9), respectively. For scenario 6, the number of animals in the training set was close to that of scenario 1 and only animals from the G9 generation of the other line. Performance data of animals from the generation and line combinations that did not contribute to the training or validation sets were removed from the analysis, but their pedigree information was kept in order to trace relationships back to the founding generation. For example, phenotypes and genotypes of animals from G0 to G3 of both lines were removed for scenario 4, since they were not part of the training or validation sets. The number of genotyped animals in the training and validation sets for the 12 scenarios are in Table 3.

**Table 3:** Number of genotyped animals in the training and validation sets for the six scenarios for the HRFI and LRFI validation sets

	HRFI		LRFI	
	Training	Validation	Training	Validation
Scenario 1	398	399	400	433
Scenario 2	1051	399	1005	433
Scenario 3	831	399	799	433
Scenario 4	859	399	825	433
Scenario 5	639	399	619	433
Scenario 6	389	399	403	433

*HRFI* high RFI line, *LRFI* low RFI line

### Accuracy and bias of genomic predictions

Usually the correlation between the vector of estimated breeding values (${\mathbf{EBV}}$) to be evaluated and the vector of true breeding values (${\mathbf{TBV}}$), ${\text{r}}\left( {{\mathbf{TBV}},{\mathbf{EBV}}} \right)$, cannot be computed. In the literature, multiple criteria have been proposed to quantify and compare prediction accuracies of genomic predictions between training and validation set structures and between prediction methods. Cross-validation approaches are often conducted based on ${\text{r}}\left( {{\mathbf{EBV}}, {\mathbf{y}}^{\varvec{*}} } \right)$, where ${\mathbf{y}}^{\varvec{*}}$ is either the vector of phenotypes adjusted for fixed effects or the vector of deregressed EBV of the validation set. Thus, a widely used criterion is ${\text{r}}\left( {{\mathbf{EBV}}, {\mathbf{y}}^{\varvec{*}} } \right)/\sqrt {{\text{h}}^{2} }$, where ${\text{h}}^{2}$ is the heritability of the trait. However, this criterion requires all the genotyped animals to have a sufficiently accurate ${\mathbf{y}}^{\varvec{*}}$ value [34]. When ${\mathbf{y}}^{\varvec{*}}$ is an adjusted phenotype of the animal’s own measurement, it suffers from the inability to adjust for the random residual effects. In the optimum situation, the expected value of the correlation would then be the square root of heritability [35]. Alternatively, using an EBV obtained from a complete dataset as the best predictor of TBV would cause autocorrelation between the reference and evaluated EBV when the training and validation sets are closely related through the pedigree, leading to higher correlations [35]. Legarra and Reverter [34] proposed to complement the cross-validation approach with a semi-parametric approach that can be used in a large number of cases, with the advantage of not requiring knowledge of the TBV or adjustment of phenotypes. The underlying assumptions of this approach are (1) the variance components are similar in the training and validation datasets, and (2) the validation set is sufficiently diverse and large (i.e. composed of various families). In brief, with their approach, the correlation between EBV using part of the dataset (partial) and EBV obtained using the whole dataset results in an estimator of the ratio of the accuracies of the EBV from these two datasets. We followed this approach to evaluate the potential for genomic prediction when including data from a related line compared to genomic prediction using all data from the target line, which will be referred to as GEBV_w_ (GEBV obtained using the whole dataset), i.e., to obtain GEBV_w_ for the validation set of each line, two separate ssGBLUP analyses were performed (one per line). GEBV_p_ (GEBV obtained using partial dataset) were the GEBV obtained from the six scenarios for the validation sets in each target line. The criterion for prediction accuracy for each trait and each scenario was then the correlation between ${\text{GEBV}}_{\text{p}}$ and ${\text{GEBV}}_{\text{w}}$, ${\text{r}}\left( {{\text{GEBV}}_{\text{p}} , {\text{GEBV}}_{\text{w}} } \right)$. Bias of the genomic predictions was computed as the deviation of the regression coefficient of ${\text{GEBV}}_{\text{w}}$ on ${\text{GEBV}}_{\text{p}}$ from 1, as also proposed in [34].

Standard errors of the prediction accuracy correlations, *r*, were obtained as $\sqrt {\left[ {\left( {1 - {\text{r}}^{2} } \right)/\left( {n - 2} \right)} \right]}$, where *n* is the number of animals used to obtain correlations in the validation sets. Differences between correlations in different scenarios were tested using the Williams t-test in the psych R package [36–38]. Significant differences between each scenario and the control scenario (scenario 1) are reported to identify the scenarios that provide prediction accuracies similar to the control scenario.

### Relationships between training and validation sets

For each scenario, the maximum, average, and minimum relationship coefficients between training and validation sets in the ${\mathbf{H}}$ matrix were computed. To distinguish the strength of relationships originating from the two lines, all three measurements were computed separately for pigs of the validation set with the subset of the training set that belonged to (1) the target line and (2) the other line. The average relationships were calculated as the mean of the off-diagonal elements of the corresponding relationship matrices for the genotyped individuals.

## Results

### Variance components

The five studied traits showed low to moderate heritabilities that ranged from 0.12 ± 0.02 (RFI) to 0.36 ± 0.05 (BFT) (Table 4). The ratio of litter effect variance to phenotypic variance (${\text{l}}^{2}$) was lower than the heritability for all traits, ranging from 0.07 ± 0.02 (FCR) to 0.12 ± 0.02 (BFT).

**Table 4:** Estimates of variance components (SE) of the studied traits

Trait	Phenotypic variance	Heritability	Litter effects^a^
ADG	5811.70 (164.75)	0.25 (0.04)	0.10 (0.02)
BFT	14.37 (0.47)	0.36 (0.05)	0.12 (0.02)
DFI	0.04 (0.001)	0.24 (0.04)	0.09 (0.02)
FCR	0.04 (0.001)	0.24 (0.04)	0.07 (0.02)
RFI	0.01 (0.004)	0.12 (0.02)	0.08 (0.02)

*ADG* average daily gain (g/day), *BFT* backfat thickness (mm), *DFI* daily feed intake (kg/day), *FCR* feed conversion ratio (kg/kg), *RFI* residual feed intake (kg/day)

^a^As a proportion of phenotypic variance

### Prediction accuracies

**Fig. 2:**
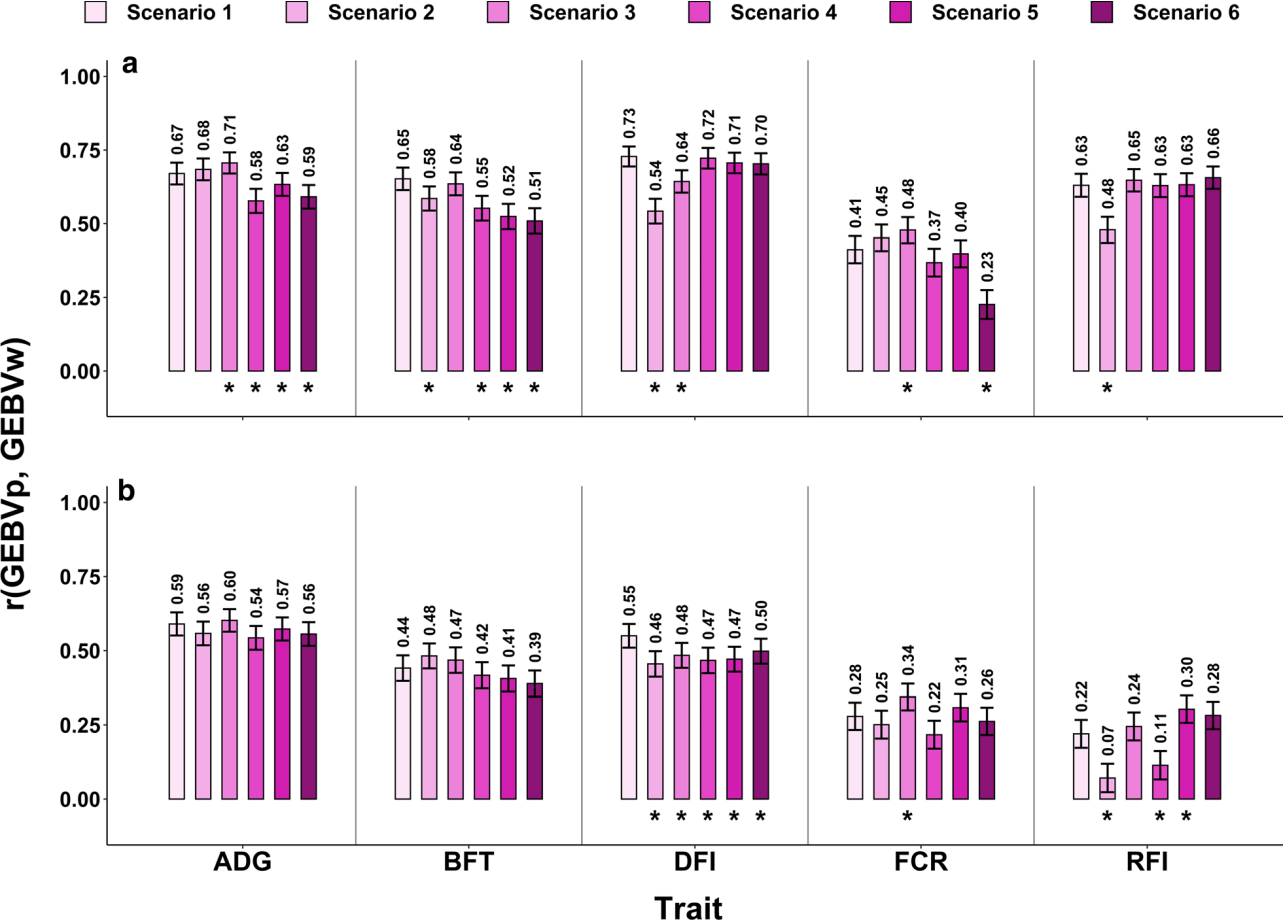
Correlations between GEBVp and GEBVw, and their SE as error bars for the HRFI (**a**) and LRFI (**b**) lines. *Significant difference with scenarios 1 (control) based on the Williams t-test at a 0.05 level. *RFI* residual feed intake, *ADG* average daily gain, *FCR* feed conversion ratio, *DFI* daily feed intake, *BFT* backfat thickness

Prediction accuracies, ${\text{r}}\left( {{\text{GEBV}}_{\text{p}} , {\text{GEBV}}_{\text{w}} } \right)$, for the different scenarios are shown in Fig. 2 for the two lines. Accuracies ranged from 0.07 to 0.73, depending on the validation line, trait, and scenario. The tested scenarios could be classified into two groups based on their design and how it affected the prediction accuracy of each trait. Removing the earlier generations of the target line from the training set (from scenarios 1, 2, 3 to scenarios 4, 5, 6) tended to decrease the prediction accuracy for ADG, BFT, and DFI, while FCR and RFI showed different patterns in response to changes in the structure of the training set.

The differences in prediction accuracies for ADG, BFT and DFI from scenario 1 to scenario 2 and 3 showed that the inclusion of different generations of the other line in the training set led to marginal changes in accuracy, with decreased correlations in most cases (BFT in the HRFI line and DFI). In scenarios 4, 5, and 6, the proportion of animals from the target line was low in the training set compared to scenarios 1, 2, and 3. This reduction generally led to a decrease in the prediction accuracies for ADG, BFT, and DFI compared to scenario 1. However, these differences in accuracy were only significant for ADG and BFT in the HRFI line and for DFI in the LRFI line.

Scenarios for FCR and RFI showed different patterns compared to the previous traits. Prediction accuracies for FCR followed a pattern similar to those of the other traits for all scenarios, except for scenario 3, which showed a 17 to 21% higher accuracy compared to scenario 1. Prediction accuracies for RFI decreased from scenario 1 to scenario 2, and scenario 1 to scenario 4 for the LRFI target line, which were the scenarios with the maximum number of individuals from the other line in the training set. In the other scenarios, the prediction accuracies for RFI were similar or higher than for scenario 1.

The prediction accuracies for FCR in all scenarios, except scenario 6, were higher for validation animals in the HRFI line than in the LRFI line. The average differences in accuracy by trait ranged from + 0.07 for ADG to + 0.40 for RFI (Fig.2).

### Prediction biases

**Fig. 3:**
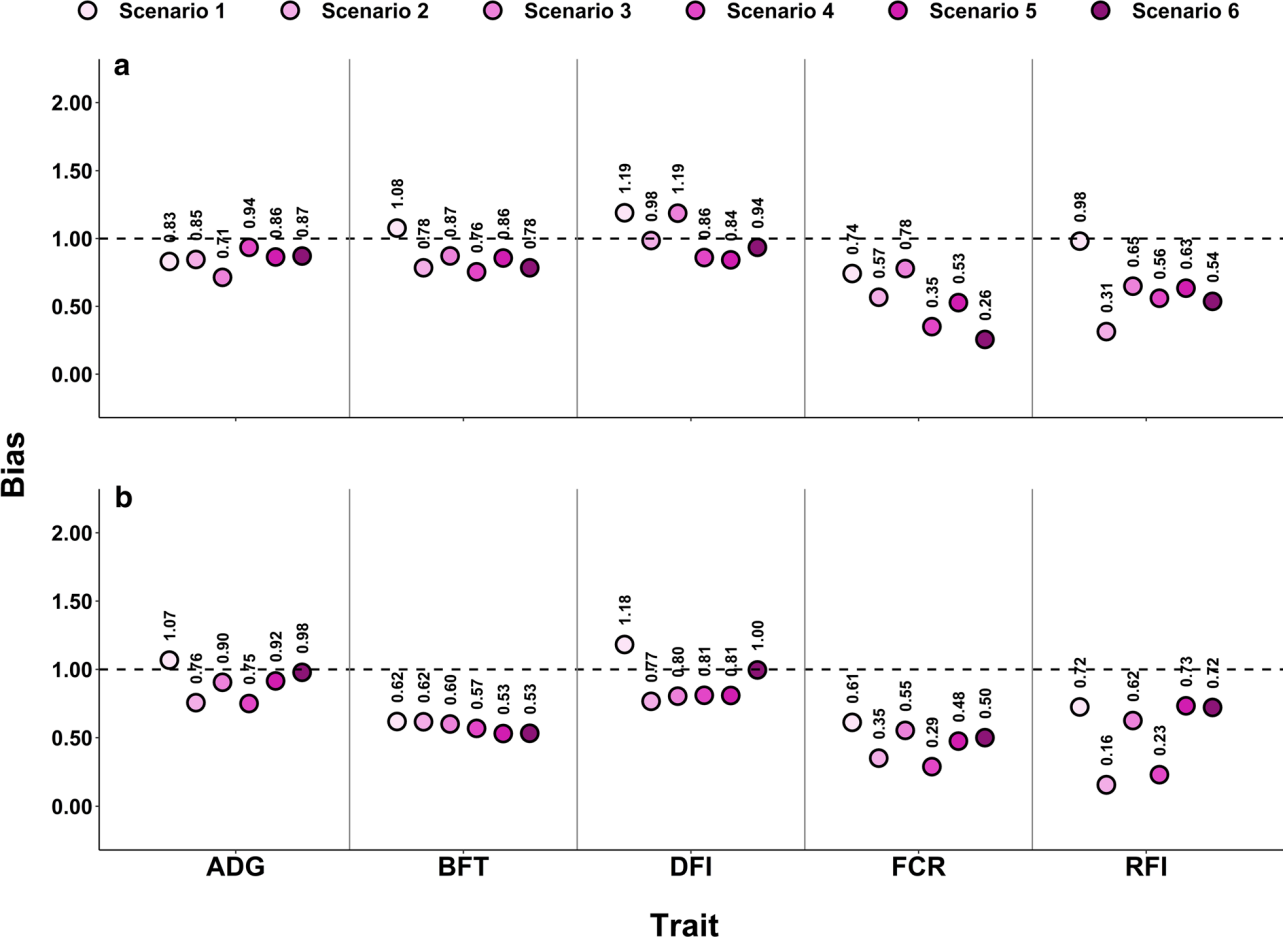
Bias (regression coefficients of GEBVw on GEBVp) for the HRFI (**a**) and LRFI (**b**) lines. *RFI* residual feed intake, *ADG* average daily gain, *FCR* feed conversion ratio, *DFI* daily feed intake, *BFT* backfat thickness

Overall, regression coefficients of ${\text{GEBV}}_{\text{w}}$ on ${\text{GEBV}}_{\text{p}}$ were consistently below 1 for FCR and RFI for both validation sets (Fig. 3). Regression coefficients for these two traits also showed more variation across the scenarios compared to ADG, BFT and DFI.

Bias for GEBV in the HRFI validation set followed the same trend, but at different magnitudes, for all traits, except ADG (Fig. 3a). On average, scenarios 1, 2, and 3 showed less biases than scenario 4, 5, and 6 for BFT, DFI, and FCR. The regression coefficient in scenario 1 was equal to 0.98 for RFI, slightly higher than 1 for BFT (1.08) and DFI (1.19), and lower than 1 for ADG (0.83) and FCR (0.74).

Prediction of GEBV for the LRFI validation set did not follow the same pattern of change across scenarios between the traits. Regression coefficients of all scenarios showed biases smaller than 1 for BFT, FCR, and RFI (Fig. 3b). Biases were smallest for DFI (scenario 6) and ADG (scenarios 1, 5 and 6). Overall, biases of GEBV for this line were moderate for scenario 6 compared to the other scenarios, except for BFT (0.53). Biases were larger for scenarios 2 and 4, compared to scenarios 5 and 6, for all traits except for BFT.

### Relationships between and within training and validation sets

**Fig. 4:**
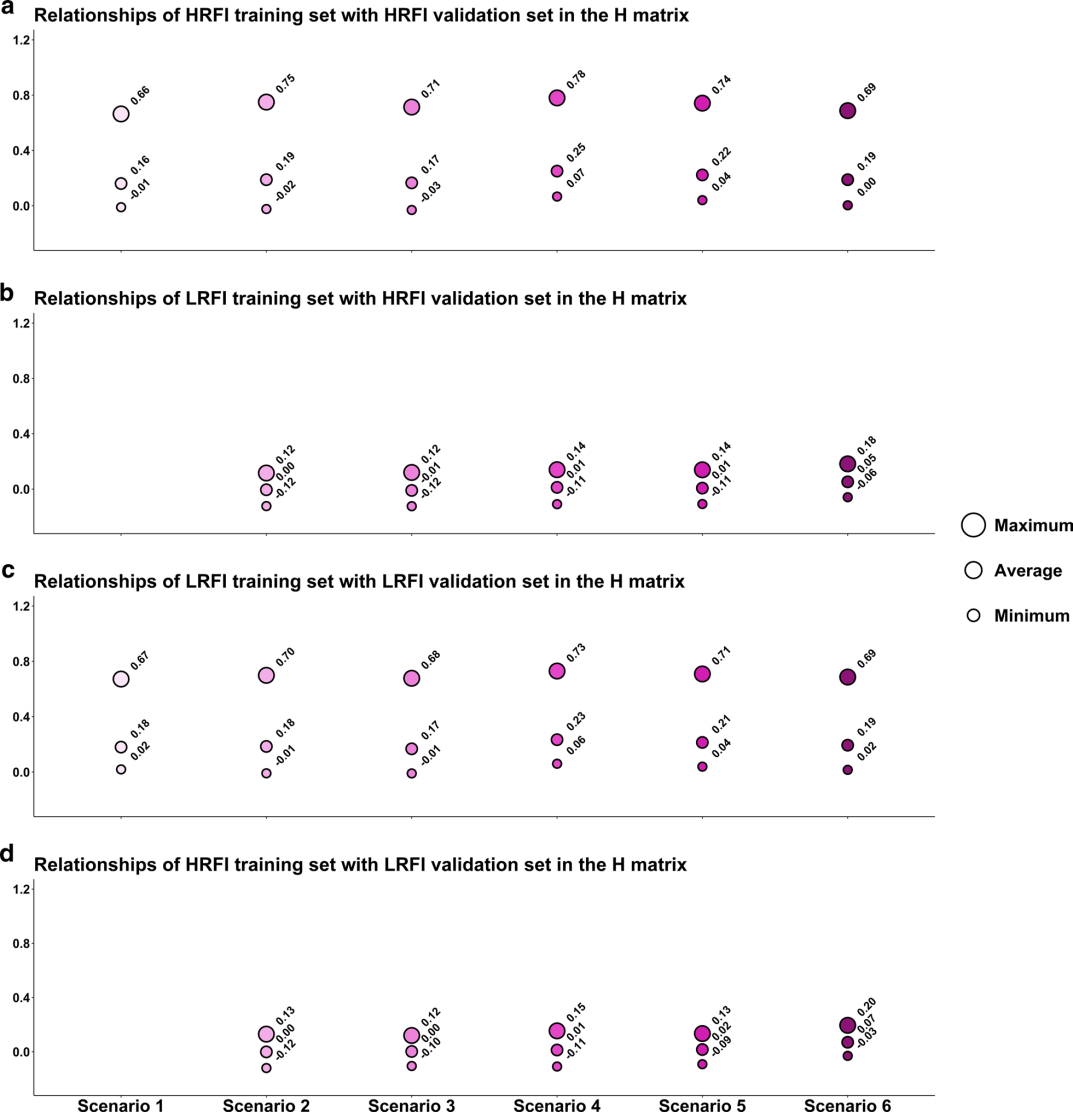
Average, minimum and maximum relationship coefficients in the H matrix between individuals of the validation set, and individuals of the training set from the target line and from the reverse line, for **a** and **b** the HRFI target line, for **c** and **d** the LRFI target line

Relationships between the validation set and the training individuals from the target line were on average higher in scenarios 4 to 6 than in scenarios 1 to 3 (Fig. 4a and c). The highest average was obtained for scenario 4 (around 0.25) and the smallest average for scenarios 1 and 3 (around 0.16 and 0.17). The maximum relationship coefficient between these two cohorts was greater than 0.66 for all scenarios, with the smallest maximum found for scenario 1 when the training set included only individuals from the target line, and the highest maximum for scenario 4 (around 0.78), when the relative number of animals from the other line in the training set was larger.

Relationship coefficients between the validation set and the training individuals of the other line were lower than those with the training individuals of the target line, but the maximum values were reached for scenario 6, i.e. equal to 0.18 and 0.20 for the HRFI and LRFI target lines, respectively (Fig. 4b and d). All other scenarios had lower maximum relationships, ranging from 0.12 to 0.15.

## Discussion

The aim of our study was to investigate different combinations of two lines derived from a common origin to evaluate the potential of building a training set for the genomic prediction of feed efficiency related traits in lines that are small or do not have much data available. Multiplying by ~ 2.5 (scenario 2), ~ 2 (scenarios 3 and 4), and ~ 1.5 times (scenario 5) the number of genotyped individuals in the training set by recruiting animals from the other line show no or little increase of prediction accuracy. This would probably not justify the additional genotyping costs involved. However, they can be considered for practical implementation of combined training sets since, in most cases, the prediction accuracies obtained in scenarios 5 and 6 were similar to those of the control scenario 1. These scenarios reflect most of the practical situations targeted in our study. Indeed, for breeding programs in small populations, phenotypic or genotypic information of individuals from earlier generations might not be available, and the sampling size in recent generations might be limited to a few hundred. Our results show that, a training population that includes recent generations of one population and data from a more distant subpopulation, could be a solution to achieve prediction accuracies similar to what would be achieved if data were available for individuals of the same population. This could even improve the prediction accuracies for traits under selection.

### Computation of prediction accuracies and biases

Variance components of the evaluated traits were estimated using the ${\mathbf{A}}$ matrix on the full dataset with both lines combined. All estimated heritabilities were in the range of values reported in the literature for these traits [8, 39–42]. Using these variance components, the accuracy of GEBV was computed for the six scenarios to predict validation animals from each line using ssGBLUP. Prediction accuracies were computed using a cross-validation method combined with a semi-parametric approach [30]. Indeed, in our case, accuracies of the adjusted phenotypes or of deregressed EBV were too low to be used in a criterion such as ${\text{r}}\left( {{\text{GEBV}}_{\text{p}} , {\text{y}}^{*} } \right)/\sqrt {{\text{h}}^{2} }$, since only two-thirds of the individuals had their own phenotype. This would result in larger standard errors of the correlations and, thus, less power to test differences between scenarios, as shown in Additional file 1: Figures S1 and S2. The underlying assumptions of the semi-parametric approach are that (1) the validation set is sufficiently diverse and large (i.e. composed of various families), and (2) variance components are similar in the training and validation datasets. The first assumption was well covered in our study, since all breeding individuals, plus some progeny of each family, were phenotyped and genotyped. The second assumption was potentially less covered, which could explain some of the biases in prediction observed. Indeed, when estimating variance components separately in the two lines, different residual variances were estimated for some traits, resulting in lower heritability estimates for DFI (24%), FCR (43%), and RFI (22%) in the LRFI line than in the HRFI line. Legarra and Reverter [30] indicated that inflation of predictions in one or the other dataset due to changes in variances can cause biased GEBV. Thus, we also tested the use of estimates of variance components from the target line for the GEBV predictions, but this resulted in increases in biases by 0.016 to 0.121 in all situations but one (results not shown). In practice, scaling the observations by the residual or phenotypic standard deviations, or accounting for the heterogeneity of residual variance across lines, could be considered to account for such differences, as proposed by Reverter et al. [43] for heterogeneous variances across herds. An alternative could be to run bivariate analyses to consider correlated traits in the two lines, instead of a single trait across the two lines. Nevertheless, in our populations, estimates of the genetic variance of RFI as the trait under selection were consistent over the nine generations in each line. Therefore, differences in observed accuracy and bias between lines could not be explained by the heterogeneity of the genetic variance over the nine generations for the trait under selection.

### Prediction accuracies for production traits

Although production traits and ssGBLUP have been discussed in the literature, few investigations have analyzed such traits in pigs with this method. Therefore, in the discussion that follows, we refer to published genomic prediction studies on these traits that often use other methods. Our objective in this part is to validate the prediction accuracies obtained with scenario 1, in which the structure of the training population is close to those of previous studies. When comparing studies, it is worth noting that ssGBLUP generally has a higher accuracy than the usual GBLUP or Bayesian approaches that use only data of genotyped animals. Thus in theory, the comparisons should favor ssGBLUP approaches. However, most previous studies were based on prediction to a single generation of candidates, which could favor higher prediction accuracies. In spite of these differences, overall, our estimates were within the range of accuracies reported in the literature, except for FCR and RFI, for which accuracies were higher in the HRFI validation set and lower in the LRFI line than those reported in the literature. In an investigation on 8113 Danish Duroc pigs with 60K imputed SNP genotyping information, an ${\text{r}}\left( {{\text{GEBV}}_{\text{p}} , {\text{y}}^{*} } \right)/\sqrt {{\text{h}}^{2} }$ of 0.41 was reported for ADG [41]. In a study with 620 commercial boars, an ${\text{r}}\left( {{\text{GEBV}}_{\text{p}} , {\text{y}}^{*} } \right)/\sqrt {{\text{h}}^{2} }$ of 0.61 was reported for BFT with ridge regression BLUP (RR-BLUP) and of 0.56 with Bayesian LASSO [39]. A similar value of 0.55 was reported for Danish Duroc pigs [41]. Zhang et al. [9] reported an ${\text{r}}\left( {{\text{GEBV}}_{\text{p}} , {\text{y}}^{*} } \right)/\sqrt {{\text{h}}^{2} }$ of 0.38 for DFI in a Duroc population using a 80K SNP chip and the GBLUP method in a design with 1167 training animals and 196 validation animals. They reported a higher accuracy (0.45) when using a 650k SNP chip and the BayesB method. Prediction accuracies of GEBV for FCR and RFI are rarely reported in the literature. Christensen et al. [8] reported a prediction accuracy of 0.16 for FCR using a bivariate ssGBLUP model. Jiao et al. [42] obtained a low prediction accuracy of 0.09 for RFI (measured as ${\text{r}}\left( {{\text{GEBV}}_{\text{p}} , {\text{y}}^{*} } \right)/\sqrt {{\text{h}}^{2} }$) using the BayesA method with 1047 training animals and 516 validation animals for the Duroc boars. Thus, overall in pig studies, prediction accuracies are low to moderate for ADG and BFT, and low for feed efficiency traits.

### Prediction accuracies depending on the training set composition

Compared to FCR and RFI, ADG, BFT, and DFI showed different changes in prediction accuracy compared to scenario 1 when the structure of the training set was changed. For ADG, BFT, and DFI, removing the earlier generations of the target line from the training set (from scenarios 2 and 3 to scenarios 4, 5 and 6) generally decreased prediction accuracy to a lesser extent. The average and maximum relationships between the validation set and the training subsets were higher in scenarios 4, 5, and 6 than in scenario 1. The maximum relationship between the validation set and the training subsets, which was previously recommended as an indicator of potential accuracies [44], was lowest in scenario 1 and highest in scenario 4, likely due to changes in allele frequencies between the early and late generations within a line. This implies that the general decrease in accuracy in the scenarios 4, 5, and 6 could be attributed neither to these changes in relationships between sets, nor to the differences in prediction accuracies between lines. Moreover, the accuracy of GEBV resulting from ssGBLUP analyses should be less sensitive to the structure of the set of genotyped animals, and accordingly, to the strength of relationships between and within training and validation sets [45] because the ${\mathbf{H}}$ matrix aggregates information from both ${\mathbf{A}}$ and ${\mathbf{A}}_{22}$. This structure of the ${\mathbf{H}}$ matrix has two major effects on the GEBV of a given animal: first, it contributes the parent average EBV of the animal using the ${\mathbf{A}}$ matrix, and second, it adjusts for the different levels of relationships of the animal with other genotyped animals using the ${\mathbf{A}}_{22}$ matrix [45, 46]. de Roos et al. [19] reported that the benefits of combining populations in a training set are greatest when the populations have diverged for only a few generations and when the heritability of the trait is low. They also showed that increasing the number of animals from a given population in the training set increased prediction accuracy in that population. Considering that de Roos et al. [19] did not include the effect of selection in their simulations, this could partly explain our results for ADG, BFT, and DFI.

### Impact of selection on accuracy and bias of predictions

The changes of accuracy across the scenarios were more diverse for RFI and FCR, with either increases or relatively similar accuracies compared to scenario 1. In some cases, the accuracy even increased as genotypes of closer generations were eliminated from the training set, which could be regarded as an effect of the different relationships between training and validation sets in these scenarios. Regarding the low prediction accuracy reported for FCR and RFI in our results and in the literature, denser SNP genotyping could probably increase the accuracy of predictions by better capturing the differences in LD between the lines. In addition, for low heritability traits, such as RFI in our study, large training populations have been reported to increase the accuracy of GEBV [47–49]. However, given that scenarios 5 and 6 resulted in accuracies that were comparable to that of the control scenario for FCR and in greater accuracies for RFI, they can be considered as optimum scenarios for an across-line genomic prediction program. Based on results from simulation, Pszczola et al. [50] declared that minimizing relationships within the reference population and maximizing them between training and validation sets maximizes the accuracy of genomic predictions. This means that including a diverse set of animals in the training set is desirable to some extent. This is consistent with our results for FCR and RFI, for which selection created two diverse sets of animals. For example, in scenario 6, including animals from G4 to G6 of the target line in the training set provided sufficient genetic links between training and validation sets, and animals from the G9 generation of the other line provided additional diversity to the training set. Overall, it seems that including animals from later generations of both lines (more diverse animals) in the training set contributed to higher accuracies of GEBV in the validation set for FCR and RFI. This might be because the SNP effects segregating in the validation set were better estimated with such a training set.

Overall, the comparison of accuracies between scenarios 4 to 6 and scenario 1 did not show an obvious effect of the removal of data of earlier generations from the training dataset. In a study using six levels of truncated data of past generations, accuracies of GEBV of young genotyped pigs were very similar for various reproductive traits [51].

### Bias of genomic predictions

Our results showed that GEBV were more biased for traits that were more affected by selection, especially when early generations of the target line were not included in the training set. The scenarios that yielded better accuracies were not those with the smaller biases, except for FCR and RFI, for which predictions were low and their regression coefficients were systematically below 1. The average and maximum relationships between training and validation sets did not affect the prediction biases in the same way for all traits, which could be due to the effect of selection. Selection in historical generations has been shown to result in considerable biases in EBV or GEBV [34, 52]. Tonussi et al. [53] emphasized that, to have accurate and unbiased GEBV with the ssGBLUP method, the ${\mathbf{G}}$ matrix should be compatible with the ${\mathbf{A}}_{22}$. Inappropriate merging of these matrices can originate from ignoring inbreeding in the structure of ${\mathbf{A}}$ and from changes in allele frequencies at QTL for the traits under selection. In our scenarios, the effect of selection in the last three generations of the validation sets was not explicitly accounted for. However, changes in marker allele frequencies in those generations were accounted for through the ${\mathbf{G}}$ matrix. Furthermore, the (co)variances used for genomic predictions were obtained from bivariate analyses including the selection criterion using the whole dataset (including validation generations). Therefore, there should be no effect of selection on the estimations of the variance components, and the prediction bias of the GEBVs should not be due to biased variance components. Computing separate accuracies and biases for sires (heavily selected) versus dams (not directly selected), could enable quantification of the effect of selection on the prediction biases. However, on the one hand, the dams had lower individual accuracies (no own phenotype), and on the other hand, only 18 sires were selected per line in these generations. Therefore, the resulting prediction accuracies and biases differed between sires and dams due to factors other than just the effect of selection and no clear conclusion could be reached. Finally, it should be mentioned that these three generations were combined into the validation set in our study to have enough individuals, but in practice, new candidates to be predicted pertain to a single unselected cohort, therefore this selection effect would be small and likely negligible.

Heritability, marker density and size of the training population have been shown to be important factors to control biases of prediction [54]. Therefore, the biases for some scenarios in this study could be explained by the low to medium heritability of the traits, the medium marker density information, and the small number of individuals in the training population. Testing similar prediction scenarios while ignoring pedigree relationships in the non-genotyped generations would lead to substantially biased predictions, especially for traits affected by selection (for instance, 1.61 for RFI predictions in the HRFI line for scenario 6). Combining full pedigree and genomic information appeared to limit bias, which is consistent with Tonussi et al. [53].

## Conclusions

The results of our study show that genomic prediction using a training set that includes animals from related lines selected in different directions could be as accurate as genomic prediction using a within-line training set. Thus, this can be a solution to create a reference set in the case of small populations, or when ancestral samples are not available at low additional costs. Combined reference sets had better prediction accuracies for traits that were highly affected by selection, which can be attributed to the inclusion of more diverse animals in the training set. Overall, among all evaluated scenarios, scenarios 5 and 6 showed optimal accuracies in most cases, which is consistent with our hypothesis that data from a related line can be used in a combined training population for genomic predictions without losing prediction accuracy. Our results also proved that absence of phenotypic records from past generations did not affect prediction accuracy but increased bias of predictions. Some of these issues could be solved by using bivariate analyses or models with heterogeneous variances to better account for changes in variances with selection in different lines. Taken together, the results of our study provide insights into the design of reference populations for small populations, particularly when lines are being developed simultaneously, which is common in poultry and pig industries, and some plant breeding plans. This strategy can be recommended to initiate a genomic selection program when historical samples are not available, or when two lines are considered and genotyping costs need to be limited.

## Supplementary information

**Additional file 1. Figure S1.** Correlation between ${\text{GEBV}}_{\text{p}}$ and ${\text{y}}^{ *}$ and their SE as bars for the HRFI (a) and LRFI (b) lines. No scenario resulted in correlations that differed from those with scenario 1 based on a Williams t-test at 5%. RFI residual feed intake, ADG average daily gain, FCR feed conversion ratio, DFI daily feed intake, BFT backfat thickness. **Figure S2.** Correlation between ${\text{GEBV}}_{\text{p}}$ and ${\text{y}}^{ *}$ divided by the square root of the heritability of corresponding traits for the HRFI (a) and LRFI (b) lines. RFI residual feed intake, ADG average daily gain, FCR feed conversion ratio, DFI daily feed intake, BFT backfat thickness.
