# Supplementary material for: The impact of training on data from genetically-related lines on the accuracy of genomic predictions for feed efficiency traits in pigs
Source: Genet Sel Evol. 2020 Oct 7;52:57. doi: 10.1186/s12711-020-00576-0 (PMC7539441; doi:10.1186/s12711-020-00576-0)
Supplement: Supplementary file 1 — Additional file 1. Figure S1. Correlation between ${\text{GEBV}}_{\text{p}}$GEBVp and ${\text{y}}^{ *}$y∗ and their SE as bars for the HRFI (a) and LRFI (b) lines. No scenario resulted in correlations that differed from those with scenario 1 based on a Williams t-test at 5%. RFI residual feed intake, ADG average daily gain, FCR feed conversion ratio, DFI daily feed intake, BFT backfat thickness. Figure S2. Correlation between ${\text{GEBV}}_{\text{p}}$GEBVp and ${\text{y}}^{ *}$y∗ divided by the square root of the heritability of corresponding traits for the HRFI (a) and LRFI (b) lines. RFI residual feed intake, ADG average daily gain, FCR feed conversion ratio, DFI daily feed intake, BFT backfat thickness. [file 12711_2020_576_MOESM1_ESM.docx]

|  |
| --- |
| **Figure S1.** Correlation between $\mathrm{GEBV}_{p}$ and $y^{*}$ and their SE as bars for the HRFI (a) and LRFI (b) lines. No scenario resulted in correlations that differed from those with scenario 1 based on a Williams t-test at 5%. RFI residual feed intake, ADG average daily gain, FCR feed conversion ratio, DFI daily feed intake, BFT backfat thickness. |

|  |
| --- |
| **Figure S2.** Correlation between $\mathrm{GEBV}_{p}$ and $y^{*}$ divided by the square root of the heritability of corresponding traits for the HRFI (a) and LRFI (b) lines. RFI residual feed intake, ADG average daily gain, FCR feed conversion ratio, DFI daily feed intake, BFT backfat thickness. |
